# From morbidity reduction to cost-effectiveness: Enhanced recovery after surgery (ERAS) society recommendations in minimal invasive liver surgery

**DOI:** 10.1007/s00423-024-03329-5

**Published:** 2024-04-23

**Authors:** Simon Moosburner, Paul M. Dahlke, Jens Neudecker, Karl H. Hillebrandt, Pia F. Koch, Sebastian Knitter, Kristina Ludwig, Can Kamali, Safak Gül-Klein, Nathanael Raschzok, Wenzel Schöning, Igor M. Sauer, Johann Pratschke, Felix Krenzien

**Affiliations:** 1 Department of Surgery, CCM | CVK, Charité – Universitätsmedizin Berlin, corporate member of Freie Universität Berlin and Humboldt-Universität Zu Berlin, Augustenburger Platz 1, 13353 Berlin, Germany; 2 Berlin Institute of Health at Charité – Universitätsmedizin Berlin, BIH Biomedical Innovation Academy, BIH Charité Clinician Scientist Program, Berlin, Germany

**Keywords:** Minimal Invasive Liver Surgery, ERAS, Cost Analysis, Complication Management

## Abstract

**Purpose:**

Minimal-invasive liver surgery (MILS) reduces surgical trauma and is associated with fewer postoperative complications. To amplify these benefits, perioperative multimodal concepts like *Enhanced Recovery after Surgery* (ERAS), can play a crucial role. We aimed to evaluate the cost-effectiveness for MILS in an ERAS program, considering the necessary additional workforce and associated expenses.

**Methods:**

A prospective observational study comparing surgical approach in patients within an ERAS program compared to standard care from 2018—2022 at the Charité – Universitätsmedizin Berlin. Cost data were provided by the medical controlling office. ERAS items were applied according to the ERAS society recommendations.

**Results:**

537 patients underwent liver surgery (46% laparoscopic, 26% robotic assisted, 28% open surgery) and 487 were managed by the ERAS protocol. Implementation of ERAS reduced overall postoperative complications in the MILS group (18% vs. 32%, *p* = 0.048). Complications greater than Clavien-Dindo grade II incurred the highest costs (€ 31,093) compared to minor (€ 17,510) and no complications (€13,893; *p* < 0.001). In the event of major complications, profit margins were reduced by a median of € 6,640.

**Conclusions:**

Embracing the ERAS society recommendations in liver surgery leads to a significant reduction of complications. This outcome justifies the higher cost associated with a well-structured ERAS protocol, as it effectively offsets the expenses of complications.

**Supplementary Information:**

The online version contains supplementary material available at 10.1007/s00423-024-03329-5.

## Introduction

Improving postoperative outcomes through multimodal patient-centered concepts such as *Enhanced Recovery after Surgery* (ERAS) has been successfully implemented for many surgical specialties [1–3]. Since 2016, liver-specific guidelines provide evidence supported measures to support all caregivers in providing the optimal perioperative care [4] and have been updated recently [5].

At the start of the ERAS program, the ERAS nurse explains the individual ERAS measures to the patient and their relatives. It is crucial in this first motivational talk to make the patient aware of their active role in the ERAS program. In addition, measures to optimize perioperative nutrition as well as a reduction in alcohol and nicotine consumption are initiated. Preoperative strategies encompass patient motivation and awareness, carbo-loading, thromboembolic prophylaxis, and the optional use of anxiolytic medication. Throughout the Intraoperative and postoperative phases, optimized anesthesia, effective pain management, promoting early mobilization and enteral nutrition play a pivotal role in ensuring a successful outcome. As one of the most important ERAS measures to reduce surgical trauma, the use of the minimally invasive technique is recommended. Adhering to a well-defined standardized pathway empowers a collaborative effort among surgeons, anesthesiologists, physiotherapists, nursing staff, and specialized ERAS nurses, resulting in a notable reduction of postoperative morbidity. This notably encompasses mitigating postoperative complications Clavien-Dindo grade I or II, such as pulmonary and cardiac complications, and reduces the overall length of hospital stay [6–8].

Our team has validated the ERAS guidelines for liver surgery for the first time, as established by the ERAS Society [6]. Through our study, we successfully demonstrated a notable reduction in the rate of complications. Specifically, we observed a decrease in overall complications from 41% in the non-ERAS group to 27% in the ERAS group. Moreover, our findings revealed that the implementation of ERAS protocols resulted in a considerable decrease in overall complications among patients receiving *minimally invasive liver surgery* (MILS). Furthermore, we have validated adherence rates and were able to demonstrate lower rates with increasing complexity of certain MILS procedures [9]. 

Offering comparable oncological radicality, MILS reduces surgical trauma and postoperative complications, especially in relation to surgical site infections. This contributes to a shorter hospital stay and leads to an improved quality of life for patients [10–14]. Given that the individual advantages of both ERAS and MILS already have been demonstrated previously with comparable improvements in postoperative complications, a critical analysis of the impact of an ERAS program with MILS on costs and potential additional benefits is imperative. This is especially relevant since both ERAS and MILS incur additional costs for healthcare providers and need to be justified within a resource-optimized environment.

## Materials and methods

**Fig. 1:**
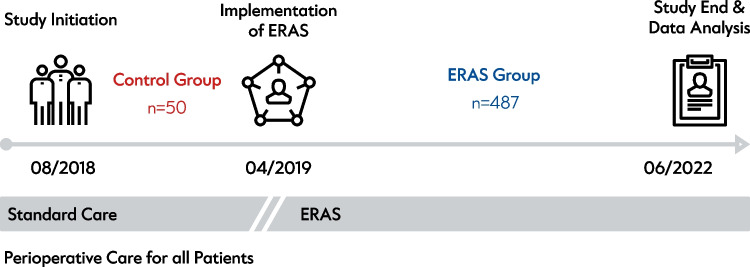
Study design. Retrospective analysis of prospectively collected data. Patients intended for liver resection were included from August 2018 and were monitored as a control group (*n* = 50) for patients receiving *Enhanced Recovery after Surgery* (ERAS) treatment after April of 2019. Analysis was terminated after June of 2022

The prospective observational study was approved by the local ethics committee under application numbers EA2/108/18 and EA4/153/18 and was registered with the German Clinical Trials Register (DRKS00030908). The study was performed following the guidelines of the Declaration of Helsinki. All study participants gave written informed consent to participate in the study and to the processing of personal data. From August 2018 to June 2022, 556 patients undergoing elective liver resection within an ERAS program at the Department of Surgery, Campus Virchow-Klinikum, Charité – Universitätsmedizin Berlin, were included (Fig. 1). Of note, this study is ongoing, and part of the data was published previously [6, 9].

Out of these patients, 50 individuals received treatment based on the clinic's standard care between July 2018 and February 2019. They were considered the control group (non-ERAS cohort) for this study. The standard care included pre-admission patient work up including blood analysis and liver function tests in case of major liver resections. Furthermore, preoperative sedative medication was allowed, if desired by the patients and intraoperatively drains were placed as desired by the surgeon. No routine low-molecular weight heparin was administered preoperatively and although early postoperative mobilization and physiotherapy was encouraged, it did not follow a stringent path. The perioperative treatment was based on the ERAS protocol of the ERAS society and documented with the *ERAS Interactive Audit System* (EIAS, Encare, Stockholm, Sweden) [6]. Following the implementation of the ERAS protocol, a total of 506 patients (487 with complete costs data) were treated from March 2019 to June 2022. These patients formed the treatment group (ERAS cohort) and were included in the study. Data on perioperative adherence and complications were recorded. Of these, 19 patients were excluded from downstream analyses due to missing cost data.

### Cost data

Total hospital costs were provided by the medical controlling office and are structured according to the *German Institute for the Hospital Remuneration System*. In Germany this is the basis of payment according to the German *diagnosis-related group* (DRG) system. Costs are bundled on a preset DRG and are calculated from admission date to discharge date. Follow-up costs such as through outpatient follow-up are not included, as they are reimbursed independently. Data are classified into the responsible department delivering therapy and respectively into personnel costs, medication, medical supply, and infrastructure costs for billing health insurances in Germany. These include the intensive care unit (ICU) and normal ward of the following departments: nephrology/dialysis, anesthesiology, endoscopy, radiology, surgery, as well as the laboratory.

### Inclusion and exclusion criteria

Patients who were at least 18 years old and underwent elective liver resection within the ERAS protocol were included. Written informed consent was obtained from all patients prior to treatment. Patients were excluded from the analysis in cases of intraoperative decision against liver resection, such as cases with peritoneal carcinosis, or in cases of simultaneous resection of another organ during the same surgery (e.g., colorectal resection).

### ERAS protocol

The applied ERAS protocol was based on the first iteration of the official ERAS guidelines for liver surgery of the ERAS Society from 2016 and was adapted according to the revised 2022 Guidelines [4, 5]. The ERAS protocol was implemented in 2019 and supervised by an ERAS core team, consisting of surgeons, anesthesiologists, physiotherapists, nursing staff and ERAS nurses [6]. Based on the ERAS measures of the EIAS, standard operating procedures as well as patient information brochures and patient diaries were created. The implementation of the ERAS protocol comprised specific interdisciplinary ERAS training of the staff and regular audit meetings.

### Statistics

Statistical analyses were performed using R (version 4.3; R Foundation for Statistical Computing, Vienna, Austria). Patients were divided into groups according to type of resection (open surgery or MILS) as well as type of perioperative care (standard care or ERAS)*.* Analysis between groups was performed using the Wilcoxon rank sum test for continuous variables and Pearson's Chi-squared test or Fisher's exact test for categorical variables. For comparison of two continuous variables, spearman´s rank correlation coefficient was calculated. The significance level (α-level) chosen was 0.05. To reduce confounding effects in chosen surgical approach and time-dependent differences since implementation of ERAS, we performed propensity score matching. ERAS/standard care and MILS/open surgery were matched for surgical approach, extent of resection, WHO performance status, previous abdominal surgery, and primary diagnosis. We matched using nearest-neighbor matching with a caliper of 0.2 with the packages *matchit* and *Matching*.

## Results

### Baseline patient and surgical characteristics

During the study period, 537 patients who underwent liver surgery had a comprehensive cost data set. Most surgeries were performed laparoscopically (46%) followed by open surgery (28%) and robotic assisted surgery (28%). Median age of patients was 63 years (range 18—89 years) and 46% were female. 487 patients were managed by the ERAS protocol, 50 patients received standard perioperative care and were the control group (Table 1). Most patients were rated *American Society of Anesthesiologists physical status classification system* (ASA) II or III (93%), had a median body mass index (BMI) of 25.5 kg/m^2^ and had no history of smoking (83%), diabetes (85%), or excessive alcohol consumption (88%). Surgery secondary to neoadjuvant chemotherapy was performed in 36% of cases and in 4% of cases after radiotherapy. Baseline patient data between patients receiving standard perioperative care (pre ERAS) or ERAS did not differ, except for neoadjuvant radiotherapy which was present in more cases in the control group, and a history of previous abdominal surgery which was more common in patients receiving ERAS. Regarding the surgical approach, MILS patients had better WHO performance statuses and more benign liver lesions.

**Table 1:** Patient Characteris tics

Variable	Overall *n* = 537^1^	Standard Care *n* = 50^1^	ERAS *n* = 487^1^	*p*-value^2^	Open *n* = 153^1^	MILS *n* = 384^1^	*p*-value^2^
Age (Years)	63 (54, 71)	67 (53, 75)	63 (54, 71)	0.2	62 (54, 72)	63 (54, 71)	> 0.9
Sex (female)	247 (46%)	21 (42%)	226 (46%)	0.6	68 (44%)	179 (47%)	0.6
BMI (kg/m^2^)	25.5 (22.5, 29.0)	26.4 (23.0, 28.7)	25.5 (22.3, 29.0)	0.7	24.9 (22.1, 28.2)	25.8 (22.5, 29.0)	0.3
ASA-classification				0.4			0.12
ASA I	30 (5.6%)	3 (6.0%)	27 (5.5%)		4 (2.6%)	26 (6.8%)	
ASA II	217 (40%)	23 (46%)	194 (40%)		61 (40%)	156 (41%)	
ASA III	285 (53%)	23 (46%)	262 (54%)		88 (58%)	197 (51%)	
ASA IV	5 (0.9%)	1 (2.0%)	4 (0.8%)		0 (0%)	5 (1.3%)	
WHO Performance Status				0.7			0.024
Asymptomatic	486 (91%)	47 (94%)	439 (90%)		133 (87%)	353 (92%)	
Symptomatic, ambulatory	48 (8.9%)	3 (6.0%)	45 (9.2%)		17 (11%)	31 (8.1%)	
Symptomatic, < 50% in bed	2 (0.4%)	0 (0%)	2 (0.4%)		2 (1.3%)	0 (0%)	
Symptomatic, > 50% in bed	1 (0.2%)	0 (0%)	1 (0.2%)		1 (0.7%)	0 (0%)	
Smoked daily before surgery	93 (17%)	10 (20%)	83 (17%)	0.6	26 (17%)	67 (17%)	> 0.9
Daily > 3 standard glasses of alcohol	64 (12%)	6 (12%)	58 (12%)	> 0.9	16 (10%)	48 (13%)	0.5
Diabetes				0.5			0.3
None	454 (85%)	40 (80%)	414 (85%)		134 (88%)	320 (83%)	
Yes, on medication	78 (15%)	10 (20%)	68 (14%)		19 (12%)	59 (15%)	
Yes, diet control	5 (0.9%)	0 (0%)	5 (1.0%)		0 (0%)	5 (1.3%)	
Primary Diagnosis				0.69			< 0.001
Primary Liver Cancer	176 (33%)	18 (36%)	158 (32%)		64 (42%)	112 (29%)	
Secondary Liver Tumor	255 (47%)	20 (40%)	235 (48%)		74 (48%)	181 (47%)	
Benign Liver Tumor	75 (14%)	8 (16%)	67 (14%)		8 (5.2%)	67 (17%)	
Other	31 (5.8%)	4 (8.0%)	27 (5.5%)		7 (4.6%)	24 (6.3%)	
Major Liver Resection	312 (58%)	24 (48%)	288 (59%)	0.13	115 (75%)	197 (51%)	< 0.001
Operative Time (min)	251 (168, 326)	262 (164, 332)	251 (169, 323)	0.8	309 (241, 386)	227 (147, 298)	< 0.001
Type of Surgery				0.14			< 0.001
Open	153 (28%)	16 (32%)	137 (28%)		153 (100%)	0 (0%)	
Laparoscopic	247 (46%)	27 (54%)	220 (45%)		0 (0%)	247 (64%)	
Robotic assisted	137 (26%)	7 (14%)	130 (27%)		0 (0%)	137 (36%)	
Type of Surgery				0.2			< 0.001
Right-sided liver surgery	142 (26%)	8 (16%)	134 (28%)		57 (37%)	85 (22%)	
Other segmentectomies and minor resections	279 (52%)	28 (56%)	251 (52%)		60 (39%)	219 (57%)	
Left-sided liver surgery	107 (20%)	14 (28%)	93 (19%)		36 (24%)	71 (18%)	
Neoadjuvant Chemotherapy	195 (36%)	16 (32%)	179 (37%)	0.5	63 (41%)	132 (34%)	0.14
Neoadjuvant Radiotherapy	23 (4.3%)	7 (14%)	16 (3.3%)	0.003	6 (3.9%)	17 (4.4%)	0.8
Previous Abdominal Surgery	233 (43%)	11 (22%)	222 (46%)	0.001	75 (49%)	158 (41%)	0.10
Preoperative bilirubin (mmol/l)	5.3 (0.7, 8.7)	7.8 (5.5, 10.3)	5.1 (0.6, 8.6)	< 0.001	6.3 (1.1, 10.9)	5.1 (0.6, 8.2)	0.005
Clavien-Dindo				0.085			< 0.001
No Complication	380 (71%)	30 (60%)	350 (72%)	*0.079*	71 (46%)	309 (80%)	< *0.001*
CD I-II	52 (9.7%)	9 (18%)	43 (8.8%)	*0.045*	25 (16%)	27 (7.0%)	< *0.001*
CD III-V	105 (20%)	11 (22%)	94 (19%)	*0.6*	57 (37%)	48 (13%)	< *0.001*
Length of Hospital Stay (days)	7 (5, 9)	7 (5, 9)	7 (5, 9)	0.8	9 (7, 17)	6 (5, 7)	< 0.001
Overall Cost (EUR)	15,348 (11,838, 21,953)	13,189 (9,972, 15,728)	15,791 (11,954, 22,431)	< 0.001	20,742 (14,859, 31,603)	14,296 (11,234, 17,911)	< 0.001
Overall Earnings (EUR)	17,668 (15,818, 22,325)	18,548 (13,677, 21,479)	17,668 (15,876, 22,325)	0.14	21,652 (17,810, 24,814)	16,915 (15,334, 21,508)	< 0.001
Overall Profits (EUR)	1,205 (-2,740, 4,765)	3,884 (-106, 7,506)	1,040 (-3,096, 4,455)	0.001	-633 (-7,058, 3,897)	1,466 (-1,795, 5,021)	< 0.001

^1^ Median (IQR); *n* (%)

^2^ Wilcoxon rank sum test; Pearson's Chi-squared test; Fisher's exact test

*ASA*, American Society of Anesthesiologists; *BMI*, Body Mass Index; *WHO*, World Health Organization, *ICU*, Intensive Care Unit

Secondary liver tumors (47%) and primary liver cancer (33%) constituted most surgical cases. Median operative time was 251 minutes and 58% were major liver resections. In total, 43% of patients had previous abdominal surgery and were overrepresented in the ERAS group (46% vs. 22%, *p* = 0.001). Furthermore, there was a slight trend towards more robotic assisted liver resections and more major liver resections after implementation of ERAS. In open liver resection, operative time was longer than in MILS, more major liver resections were performed, and more malignant diagnosis were the reason for surgery. The rate of previous abdominal surgery was equally distributed here.

### Reduction of perioperative complications through MILS in combination with an ERAS program

**Fig. 2:**
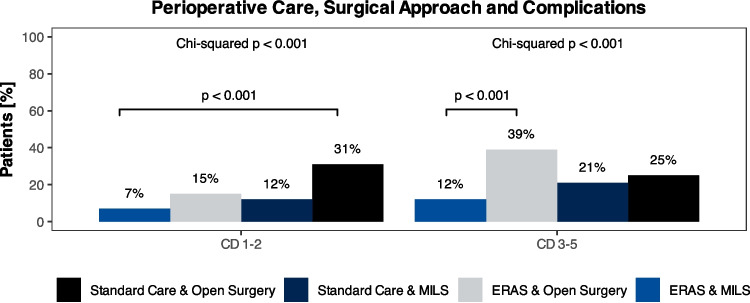
Bar plot comparing the proportions of moderate, Clavien-Dindo (CD) I-II, and severe complications (CD III-V) depending on surgical technique (minimal invasive liver surgery – MILS vs. open surgery) and perioperative surgical care (Enhanced Recovery after Surgery – ERAS vs. standard care)

Overall complication rate was 29%. ERAS reduced Clavien-Dindo Grade I and II complications (18% vs. 8.8%; *p* = 0.045) but had no effect on severe complications. In the matched subgroup, there remained a trend to fewer overall postoperative complications (40% vs. 16%**,** *p* = 0.022). MILS patient had significantly reduced postoperative complications compared to open surgery, regardless of severity (all *p* < 0.001). In subgroup analysis, ERAS patients who underwent MILS had the lowest overall complication rate (18% vs. 32%, *p* < 0.001) as well as a lower rate of mild (Clavien-Dindo I-II; 7% vs. 15%, *p* = 0.29) and severe complications (Clavien-Dindo III-IV; 12% vs. 39%, *p* < 0.001), when compared to ERAS patients undergoing open surgery (Fig. 2).

### Reduced complications are associated with decreased costs

**Fig. 3:**
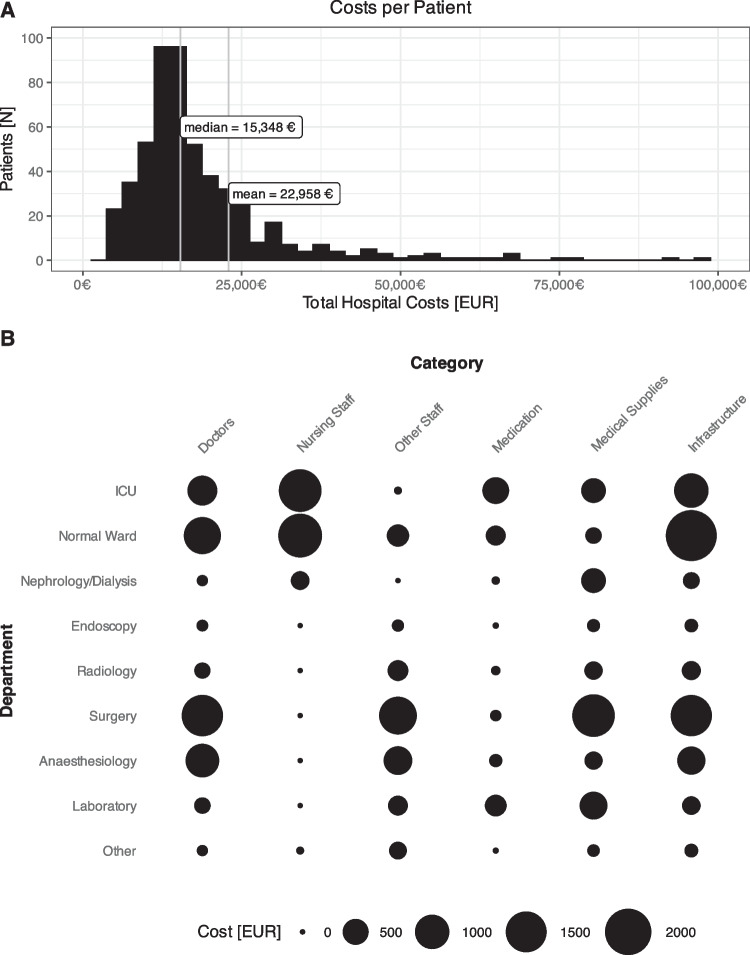
**A** Histogram and median and mean hospital costs. **B** Balloon plot of detailed costs per hospital department and resource category. The size of the circle indicates the cost in EUR (€)

**Fig. 4:**
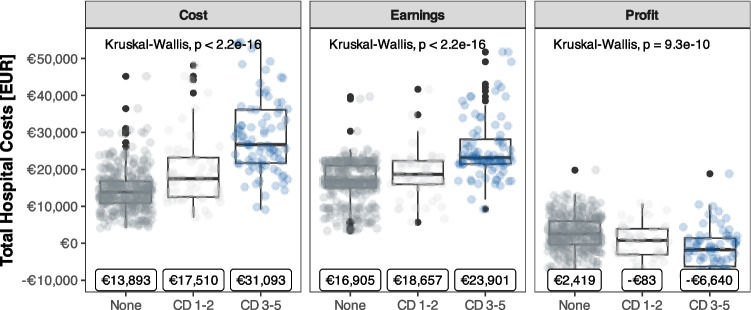
Costs, earnings and profits and their association with postoperative complications classified by Clavien-Dindo (CD). Group were created for no complications, grade I&II and grade III-V. Severity of complication increased overall costs and reduced profits. Ordinate axis is limited to € 50,000 for improved visualization. Median values are shown at the bottom

Overall median cost per surgical case was € 15,348 and generated € 1,205 profit **(**Fig. 3). Complications greater than Clavien-Dindo II incurred the highest costs (€ 31,093) compared to minor complications (€ 18,3657) and no complications (€ 13,893; *p* < 0.001). Despite increased compensation in the event of major complications, from € 16,905 for no complications to € 23,901 in cases with severe complications, profit margins were reduced by a median of € 6,640 (*p* < 0.001, Fig. 4). The most substantial additional median costs accumulated in the intensive care unit (€ 2,440, *p* < 0.001), normal ward (€ 6,435, *p* < 0.001), and radiology department (€ 1,392, *p* < 0.001, SupplementaryTable 1).

### Fewer complications reduce length of hospital stay and associated costs

**Fig. 5:**
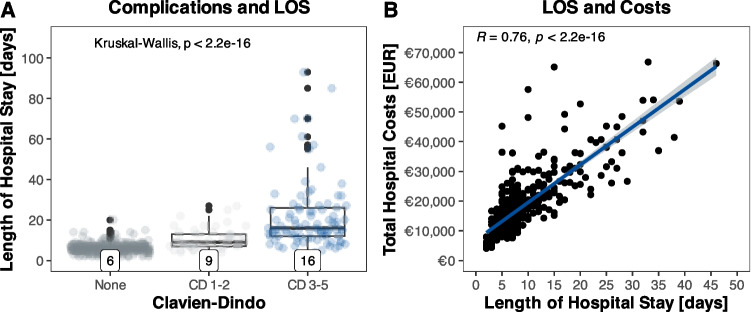
**A** Complications classified by Clavien-Dindo (CD), and length of hospital stay (LOS). Higher grade of complication led to longer hospital stays. **B** Correlation of LOS and costs. Increased LOS was associated with significantly more costs. Ordinate axis is limited to € 70,000 for improved visualization. Median values are shown at the bottom

Median hospital stay was 7 days (IQR 5–9 days) for all patients and did not change after implementation of ERAS. However, patients after MILS had a 3 day shorter hospital stay (9 vs. 6 days; *p* < 0.001) and this effect remained after propensity score matching. In cases of postoperative complications, length of hospital stays (LOS) increased significantly (*p* < 0.001) from 6 days for no complications, to 9 days for minor complications and 16 days for severe complications (Fig. 5). LOS correlated well with overall costs (spearman´s R = 0.76; *p* < 0.001).

### Detailed cost analysis

When adjusting for extent of resection and surgical technique through propensity score matching, overall cost and profits did not differ between ERAS and standard perioperative care (Table 2). MILS cases generated more profit and were attributed to lower overall cost even after matching. In detailed analysis of all involved departments and personnel or material costs, the largest cost factor was the surgery itself (€ 5,684) followed by the normal ward (€ 4,099). Salaries for doctors and other staff combined with infrastructure costs were the largest cost attributing factors. Medical supplies in the operating room were the second largest subgroup (€ 1,255). Most expensive departments outside of surgery were anesthesiology and the intensive care unit (ICU, Fig. 3).

**Table 2:** Patient Characteristics (matched)

Variable	Standard Care *n* = 50^1^	ERAS *n* = 50^1^	*p*-value^2^	Open *n* = 118^1^	MILS *n* = 118^1^	*p*-value^2^
Age (Years)	67 (53, 75)	66 (54, 75)	0.8	61 (53, 72)	65 (55, 71)	0.4
Sex (female)	21 (42%)	22 (44%)	> 0.9	51 (43%)	45 (38%)	0.5
BMI (kg/m^2^)	26.4 (23.0, 28.7)	25.9 (22.5, 28.2)	0.8	25.1 (22.4, 29.0)	26.3 (22.3, 29.4)	0.4
ASA-classification			0.8			0.5
ASA I	3 (6.0%)	2 (4.0%)		4 (3.4%)	6 (5.1%)	
ASA II	23 (46%)	21 (42%)		48 (41%)	44 (37%)	
ASA III	23 (46%)	27 (54%)		66 (56%)	66 (56%)	
ASA IV	1 (2.0%)	0 (0%)		0 (0%)	2 (1.7%)	
WHO Performance Status			0.3			0.7
Asymptomatic	47 (94%)	43 (86%)		108 (92%)	105 (89%)	
Symptomatic, ambulatory	3 (6.0%)	7 (14%)		10 (8.5%)	13 (11%)	
Symptomatic, < 50% in bed	0 (0%)	0 (0%)		0 (0%)	0 (0%)	
Symptomatic, > 50% in bed	0 (0%)	0 (0%)		0 (0%)	0 (0%)	
Smoked daily before surgery	10 (20%)	10 (20%)	> 0.9	17 (14%)	20 (17%)	0.7
Daily > 3 standard glasses of alcohol	6 (12%)	8 (16%)	0.8	15 (13%)	14 (12%)	> 0.9
Diabetes			0.4			0.10
None	40 (80%)	43 (86%)		103 (87%)	93 (79%)	
Yes, on medication	10 (20%)	6 (12%)		15 (13%)	23 (19%)	
Yes, diet control	0 (0%)	1 (2.0%)		0 (0%)	2 (1.7%)	
Primary Diagnosis			0.3			> 0.9
Primary Liver Cancer	18 (36%)	17 (34%)		36 (31%)	36 (31%)	
Secondary Liver Tumor	20 (40%)	28 (56%)		69 (58%)	69 (58%)	
Benign Liver Tumor	8 (16%)	5 (10%)		7 (5.9%)	7 (5.9%)	
Other	4 (8%)	0 (0%)		6 (5.1%)	6 (5.1%)	
Major Liver Resection	24 (48%)	33 (66%)	0.11	81 (69%)	80 (68%)	> 0.9
Operative Time (min)	262 (164, 332)	267 (209, 360)	0.2	290 (227, 360)	265 (181, 325)	0.018
Type of Surgery			0.022			< 0.001
Open	16 (32%)	13 (26%)		118 (100%)	0 (0%)	
Laparoscopic	27 (54%)	18 (36%)		0 (0%)	81 (69%)	
Robotic assisted	7 (14%)	19 (38%)		0 (0%)	37 (31%)	
Type of Surgery			0.3			0.5
Right-sided liver surgery	8 (16%)	13 (26%)		38 (32%)	31 (26%)	
Other segmentectomies and minor resections	28 (56%)	28 (56%)		54 (46%)	54 (46%)	
Left-sided liver surgery	14 (28%)	9 (18%)		26 (22%)	32 (27%)	
Neoadjuvant Chemotherapy	16 (32%)	16 (32%)	> 0.9	58 (49%)	57 (48%)	> 0.9
Neoadjuvant Radiotherapy	7 (14%)	1 (2.0%)	0.059	5 (4.2%)	9 (7.6%)	0.4
Previous Abdominal Surgery	11 (22%)	5 (10%)	0.2	68 (58%)	63 (53%)	0.6
Preoperative bilirubin (mmol/l)	7.8 (5.5, 10.3)	6.6 (5.0, 9.1)	0.14	5.6 (0.8, 9.6)	5.5 (3.8, 8.9)	0.5
Clavien-Dindo			0.022			< 0.001
No Complication	30 (60%)	42 (84%)	*0.013*	64 (54%)	99 (84%)	< *0.001*
CD I-II	9 (18%)	2 (4.0%)	*0.051*	23 (19%)	4 (3.4%)	< *0.001*
CD III-V	11 (22%)	6 (12%)	*0.3*	31 (26%)	15 (13%)	*0.013*
Length of Hospital Stay (days)	7 (5, 9)	7 (6, 9)	0.5	8 (7, 14)	7 (6, 8)	< 0.001
Overall Cost (EUR)	13,189 (9,972, 15,728)	15,851 (12,510, 23,168)	0.002	18,319 (13,421, 23,998)	14,286 (11,872, 17,359)	< 0.001
Overall Earnings (EUR)	18,548 (13,677, 21,479)	21,644 (17,402, 22,325)	0.002	21,203 (17,064, 22,325)	19,792 (16,250, 22,325)	0.047
Overall Profits (EUR)	3,884 (-106, 7,506)	3,457 (-2,099, 6,877)	0.3	1,327 (-3,652, 5,547)	3,989 (262, 7,148)	0.003

^1^ Median (IQR); *n* (%)

^2^ Wilcoxon rank sum test; Pearson's Chi-squared test; Fisher's exact test

*ASA*, American Society of Anesthesiologists; *BMI*, Body Mass Index; *WHO*, World Health Organization, *ICU*, Intensive Care Unit

Normal ward costs were lower in the MILS group (€ 3,710 vs. € 6,097; *p* < 0.001) but slightly higher in the ERAS group (€ 4,091 vs. € 3,004; *p* < 0.001). This did not change after propensity score matching. ICU costs were similar for ERAS and standard care but significantly lower in the MILS group (€ 712 vs. € 1,278; *p* < 0.001, SupplementaryFigs. 1& 2). After matching this effect was not as pronounced (€ 677 vs. € 915 for open resection; *p* < 0.001). Further cost driving factors were intraoperative medical supplies in the MILS group (€ 2,853 robotic assisted vs. € 1,219 laparoscopic (MILS € 1,641) vs. € 767 open surgery; *p* < 0.001, SupplementaryTable 1). However, costs for doctors (€ 1,777 vs. € 1,248; *p* < 0.001) were significantly lower in the MILS group.

## Discussion

MILS and ERAS are supposed to improve patient outcomes in surgery. However, the implementation of both measures involves initial infrastructure and administrative costs, along with the necessity of skilled personnel. We herein analyze the first three years after implementation of a standardized ERAS protocol for liver surgery according to the ERAS guidelines of the ERAS society. Our study demonstrates not only a further reduction in the rate of complications when combining ERAS and MILS, but also emphasize that costs increase disproportionately in the presence of postoperative complications. We contend that while implementing MILS within a robust ERAS program incurs additional costs, these expenses are offset by the significant reduction in postoperative complication rates, making it a prudent and cost-effective approach in the long run.

In our study, patients managed under the ERAS protocol did not significantly differ from those receiving standard perioperative care in terms of baseline characteristics. However, it is worth noting that the pre ERAS group had a higher prevalence of neoadjuvant radiotherapy, while the ERAS group showed a more common history of previous abdominal surgery. MILS patients demonstrated better WHO performance statuses and presented with more benign liver lesions, indicating technical limitations for resectability in MILS. It is essential to highlight that MILS is an integral part of the ERAS guidelines and strongly recommended whenever feasible.

Our data revealed a notable reduction in overall perioperative complications with the implementation of the ERAS protocol. Specifically, ERAS was associated with a significant decrease in Clavien-Dindo Grade I and II complications, in line with our previous publication covering a shorter time period [6]. Notably, when MILS and ERAS were combined, the most favorable outcomes were observed, with the lowest overall complication rate, fewer mild complications, and substantially fewer severe complications compared to patients not following the ERAS protocol. While we did not analyze time-depended effects of the ERAS implementation at out clinic, Hung et al. have previously reported at least 31 patients to be required to implement ERAS for colorectal surgery [15]. Furthermore Pisarska et al. have shown an increase followed by a slight drop and steady-state in adherence to the ERAS protocol over a 4-year period [16]. Indeed we have shown in our own clinic, that lower adherence is also associated with higher complexity of MILS, suggesting more individualized approaches for some patients [9]. As the analyzed timeframe in this cohort was 4 years, we assume to have reached a steady state of adherence, analogous to Hung et al. [15].

Open surgery combined with ERAS showed a higher proportion of Clavien-Dindo Grade III—V complications (39%). We assume this difference is due to the high proportion of biliary-enteric anastomosis in this group (45% vs. 25%). Furthermore, the exclusion of patients with biliary-enteric anastomosis (36 cases) from the analysis demonstrated similar Clavien-Dindo Grade III—V complication rates in open surgery performed with ERAS (28%) compared to standard perioperative care (25%). For MILS, there remained an 11% lower rate (12%) in combination with ERAS compared to standard perioperative care even after excluding all cases requiring biliary-enteric anastomosis. This is in line to our previous analysis [6].

The economic analysis demonstrated that complications greater than Clavien-Dindo Grade II incurred the highest costs, significantly exceeding those associated with no or minor complications. While severe complications resulted in prolonged hospital stays and necessitated additional interventions, they had a limited impact on the overall financial outcome, leading to a substantial reduction in the profit margin. Therefore, there is a clear financial incentive for hospitals to minimize complications through the implementation of MILS or even an ERAS protocol.

*Joliat *et al*.* analyzed the costs of implementing ERAS for liver surgery in a small Swiss population and found a non-significant cost reduction of about € 3,000 per patient [17]. However, laparoscopic liver resection was performed in only one out of every four patients. The biggest effect was seen in choosing MILS, which reduced ICU costs by about € 500 per case. The length of hospital stays correlated well with overall costs. Shorter hospital stays associated with fewer complications resulted in reduced costs and suggest potential cost savings when implementing MILS and ERAS protocols.

Upon detailed analysis, surgery emerged as the most substantial cost factor, followed by normal ward expenses and infrastructure costs. Notably, in the surgery category, medical supplies in the operating room were also a significant cost driver. However, the overall profit was higher in the MILS group, suggesting that MILS may have potential cost-saving benefits compared to open surgery, if patients are adequately selected. Furthermore, costs associated with doctors were lower in the MILS group. This might be attributed to fewer surgeons being required for the same surgery performed minimal invasive (especially if robotically assisted) than in open surgery. If these results are replicated in similar study cohorts, this could have far-reaching consequences for staffing levels in the future. We want to point out, that even if this trend were to be proven, less surgeons required in the operating theatre does not necessarily mean less required surgeons overall. Growing healthcare demand worldwide due to improved access to healthcare and economic growth will result in increasing patient numbers and healthcare usage [18, 19]. We would postulate a change in scope of work or higher frequency of surgeries, both requiring ample personnel to ensure patient safety.

Several studies have already demonstrated the advantageous effect of ERAS on reducing postoperative complication rates after liver resection. Our center was the first to specifically followed and investigate the ERAS guidelines for liver surgery established by the ERAS society, assessing the real-world adherence to these recommended measures [6].

In a previous meta-analysis with of over 700 patients conducted by *Ni *et al*.*, evidence was presented for the efficacy of ERAS in liver surgery. The study demonstrated a reduction in time to first flatus and to grade I and II complications [8]. This finding aligns with data from our center as well as from various centers across the globe, including those in China and Brazil. Interestingly, these results remain consistent despite the lack of homogeneity in the definition of ERAS or strict adherence to guidelines [6, 7, 20, 21].

A similar benefit has been shown for MILS, which has been proven to be equally safe and feasible as open surgery [12–14]. Recent data on the feasibility of robotic assisted liver surgery and an ERAS program by *Xie *et al*.* showed a synergistic effect in a population of 171 patients with the shortest length of hospital stay and fewest complications [22]. Furthermore, similar to our baseline cohort, costs through robotic surgery (i.e., material and infrastructure costs) were higher overall.

Comparing cost data across healthcare system can be quite challenging, as different methods for reimbursement calculation are chosen. In Germany, costs are bundled in a diagnosis-related group system (DRG). One of the main incentives is to reduce length of hospital stay as this can increase profits to an extent. Therefore, LOS itself is not a reliable parameter for patient outcome and is additionally affected by preexisting conditions of patients, socioeconomic status, and type of insurance. LOS might therefore be more an indicator of healthcare policies rather than surgical outcomes. Furthermore, we did not include outpatient costs pre-admission and after discharge, as not all costs incur to the treating hospital but can be covered by other outpatient providers, limiting the comparability. Finally, diagnosing and classifying postoperative complications is encouraged as this can lead to a case being classified as being “more complex” and hence again generating more profit [23, 24]. However, we saw negative profit margins in cases of severe complications and no adequate reimbursement for such complex cases. This system is therefore not without flaws and lawmakers are currently changing the reimbursement procedure in Germany. It will be interesting to see these changes affect patient outcomes and hospital-associated costs.

Our study is limited by the single-center design and the retrospective analysis of prospectively collected data. We had more grade III-V complications in patients with ERAS and open surgery compared to standard care and open surgery – most likely due to the complexity of the procedures in this subgroup. The implementation of ERAS coincided with the start of a robotic liver resection program, causing more robotic assisted surgeries to be performed within the ERAS period than before. Furthermore, the decision for MILS or open resection was at the discretion of the responsible department, hence more complex surgeries were mostly performed open. Despite our best efforts through propensity-score matching to alleviate these differences, a degree of uncertainty remains. However, we want to highlight the overall large sample size, low level of missingness in the data and the in-depth cost data which is available due to the nature of reimbursement in Germany. 

## Conclusion

ERAS and MILS demand a greater number of skilled personnel and can present significant initial investment costs and logistical challenges. Though these investment costs may not be directly accounted for in this analysis, they hold utmost relevance in the overall evaluation. Depending on the surgical center’s case volume, these costs will eventually yield returns over time.

However, the demonstrated advantages concerning postoperative morbidity should encourage surgical centers to not only implement ERAS *or* MILS but to combine both approaches. The associated costs are effectively offset by the reduction in postoperative complications, making it not only a medical necessity but also a financially sound and beneficial decision.

### Supplementary Information

Below is the link to the electronic supplementary material.

Supplementary file1 (PDF 500 KB)

## Data Availability

Patient data were collected as part of an observational study. Raw data for this dataset are not publicly available to preserve individuals’ privacy under the European General Data Protection Regulation.
