# Supplementary material for: From morbidity reduction to cost-effectiveness: Enhanced recovery after surgery (ERAS) society recommendations in minimal invasive liver surgery
Source: Langenbecks Arch Surg. 2024 Apr 23;409(1):137. doi: 10.1007/s00423-024-03329-5 (PMC11039530; doi:10.1007/s00423-024-03329-5)
Supplement: Supplementary file 1 — Supplementary file1 (PDF 500 KB) [file 423_2024_3329_MOESM1_ESM.pdf]

Langenbeck´s Archives of Surgery: Supplementary Material

From Morbidity Reduction to Cost-Effectiveness: Enhanced Recovery after Surgery (ERAS) in Minimal Invasive Liver Surgery

Simon Moosburner[1,2], Paul M. Dahlke[1], Jens Neudecker[1], Karl H. Hillebrandt[1,2], Pia F. Koch[1], Sebastian Knitter[1], Kristina Ludwig[1], Can Kamali[1], Safak Gül-Klein[1], Nathanael Raschzok[1,2], Wenzel Schöning[1], Igor M. Sauer[1], Johann Pratschke[1], Felix Krenzien[1,2]

1. Charité – Universitätsmedizin Berlin, corporate member of Freie Universität Berlin and Humboldt-Universität zu Berlin, Department of Surgery, Berlin, Germany
2. Berlin Institute of Health at Charité – Universitätsmedizin Berlin, BIH Academy, Clinician Scientist Program, Berlin, Germany

Corresponding Author: Priv.-Doz. Dr. med. Felix Krenzien, MD, Department of Surgery, CCM I CVK, Charité – Universitätsmedizin Berlin, Augustenburger Platz 1, 13353 Berlin (Germany), E-Mail: felix.krenzien@charite.de

Supplementary Figure 1

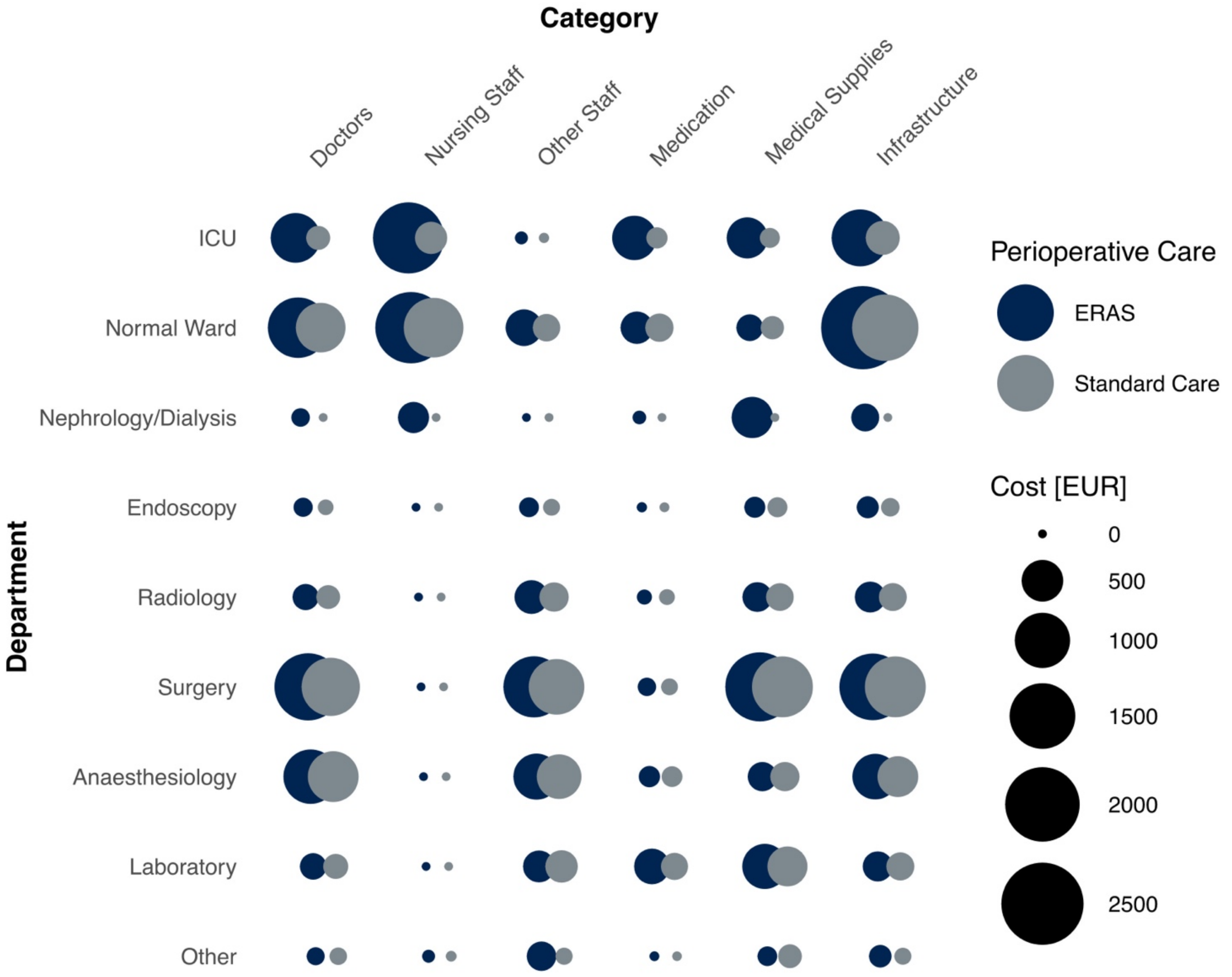

Supplementary Figure 1. Balloon plot of detailed costs per hospital department and resource category. The size of the circle indicates the cost in EUR (€) and the color indicate the type of perioperative care received.

Supplementary Figure 2

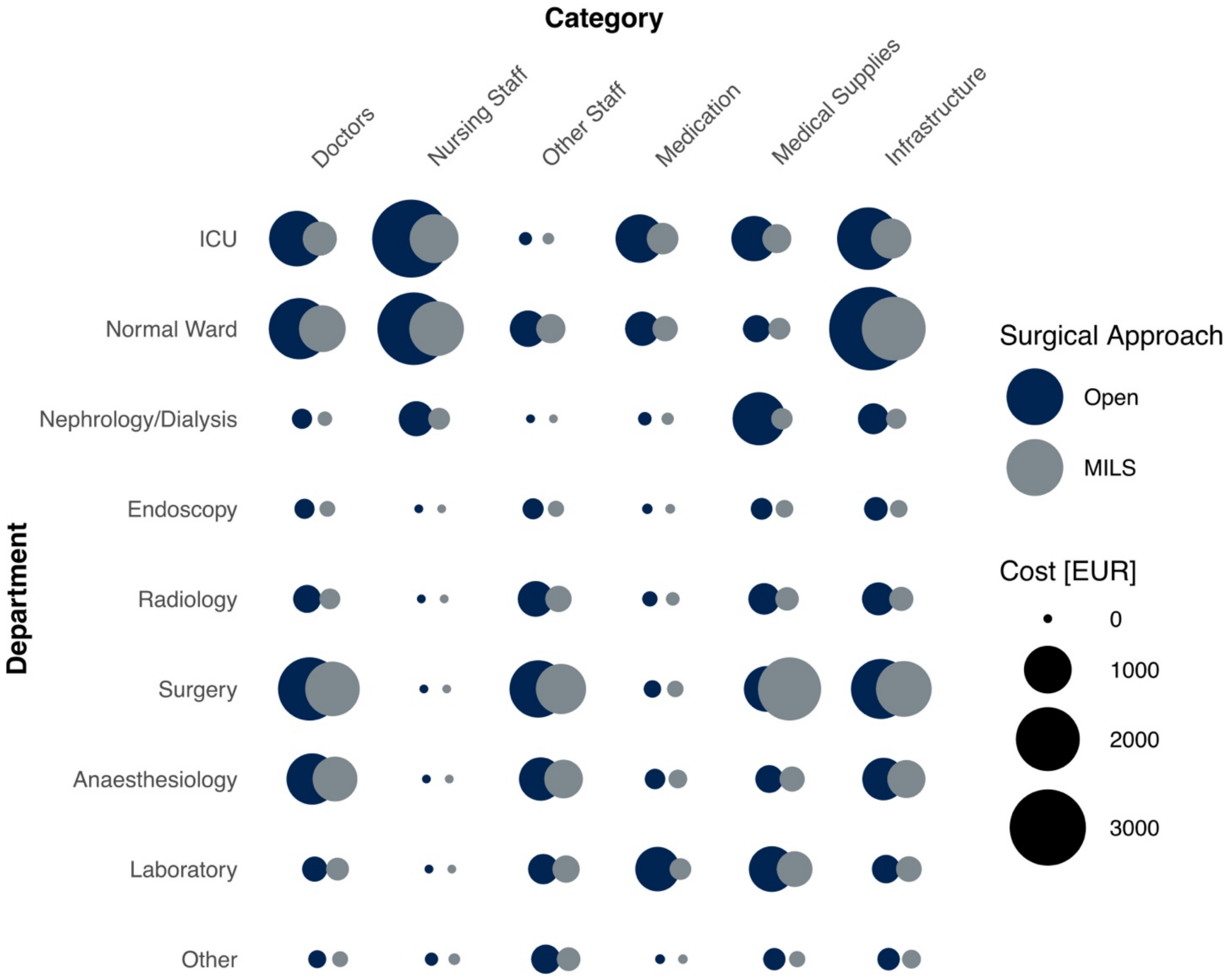

Supplementary Figure 2. Balloon plot of detailed costs per hospital department and resource category. The size of the circle indicates the cost in EUR (€) and the color indicate the type of surgical approach.

Supplementary Table 1.

Variable	Overall n = 537[1]	Standard Care n= 50[1]	ERAS n = 487[1]	p-value[2]	Open n = 153[1]	MILS n = 384[1]	p-value[2]
**Departments (Overall Costs)**							
Normal Ward	4,009 (3,220, 6,262)	3,004 (2,408, 4,660)	4,091 (3,296, 6,430)	<0.001	6,097 (3,906, 11,192)	3,710 (3,050, 4,844)	<0.001
Intensive Care Unit	799 (0, 1,758)	629 (532, 729)	841 (0, 2,028)	0.006	1,278 (709, 3,736)	712 (0, 1,012)	<0.001
Nephrology/Dialysis	0	0	0	0.2	0	0	0.039
Surgery	5,684 (4,036, 7,381)	4,393 (3,261, 5,906)	5,765 (4,115, 7,544)	<0.001	5,835 (4,294, 7,342)	5,589 (3,741, 7,386)	0.2
Anesthesiology	2,227 (1,693, 2,885)	2,189 (1,522, 2,547)	2,239 (1,715, 2,925)	0.12	2,735 (2,176, 3,253)	2,113 (1,589, 2,568)	<0.001
Endoscopy	0 (0, 0)	0 (0, 0)	0 (0, 0)	0.6	0 (0, 0)	0 (0, 0)	0.004
Radiology	121 (49, 638)	212 (121, 678)	117 (49, 636)	0.065	418 (97, 1,727)	69 (49, 366)	<0.001
Laboratory	989 (660, 1,538)	894 (532, 1,528)	997 (678, 1,542)	0.2	1,518 (955, 2,309)	869 (601, 1,271)	<0.001
**Intensive Care Unit**							
Doctors	109 (0, 267)	76 (61, 127)	112 (0, 275)	0.023	177 (101, 592)	100 (0, 139)	<0.001
Nursing Staff	260 (0, 624)	204 (151, 243)	274 (0, 675)	0.003	417 (219, 1,369)	232 (0, 355)	<0.001
Other Staff	1 (0, 2)	0 (0, 0)	1 (0, 2)	<0.001	2 (0, 5)	0 (0, 2)	<0.001
Medication	18 (0, 61)	40 (12, 70)	17 (0, 59)	0.001	47 (10, 200)	0 (0, 36)	<0.001
Medical Supplies	64 (0, 148)	44 (31, 55)	70 (0, 173)	0.001	124 (55, 326)	54 (0, 109)	<0.001
Infrastructure	318 (0, 607)	261 (169, 298)	333 (0, 686)	0.006	491 (285, 1,201)	286 (0, 405)	<0.001

**Normal Ward**							
Doctors	793 (642, 1,241)	566 (468, 899)	814 (662, 1,251)	<0.001	1,215 (813, 2,408)	735 (619, 945)	<0.001
Nursing Staff	1,084 (791, 1,870)	915 (679, 1,293)	1,100 (804, 1,907)	0.010	1,708 (1,043, 3,011)	996 (751, 1,493)	<0.001
Other Staff	238 (176, 347)	118 (99, 177)	249 (194, 361)	<0.001	329 (233, 648)	222 (166, 286)	<0.001
Medication	149 (101, 223)	117 (92, 195)	153 (102, 228)	0.022	207 (136, 427)	128 (92, 188)	<0.001
Medical Supplies	86 (65, 152)	71 (52, 111)	88 (66, 154)	0.003	136 (88, 258)	77 (61, 115)	<0.001
Infrastructure	1,650 (1,312, 2,539)	1,144 (894, 1,634)	1,685 (1,359, 2,633)	<0.001	2,472 (1,618, 4,435)	1,537 (1,255, 1,960)	<0.001
**Dialysis**							
Doctors	0	0	0	0.2	0	0	0.040
Nursing Staff	0	0	0	0.2	0	0	0.040
Other Staff	0 (0%)	0 (0%)	0 (0%)		0 (0%)	0 (0%)	
Medication	0	0	0	0.2	0	0	0.040
Medical Supplies	0	0	0	0.2	0	0	0.038
Infrastructure	0	0	0	0.2	0	0	0.040
**Endoscopy**							
Doctors	0 (0, 0)	0 (0, 0)	0 (0, 0)	0.6	0 (0, 0)	0 (0, 0)	0.002
Nursing Staff	0 (0%)	0 (0%)	0 (0%)		0 (0%)	0 (0%)	
Other Staff	0 (0, 0)	0 (0, 0)	0 (0, 0)	0.5	0 (0, 0)	0 (0, 0)	0.002
Medication	0.00 (0.00, 0.00)	0.00 (0.00, 0.00)	0.00 (0.00, 0.00)	0.4	0.00 (0.00, 0.00)	0.00 (0.00, 0.00)	<0.001
Medical Supplies	0 (0, 0)	0 (0, 0)	0 (0, 0)	0.6	0 (0, 0)	0 (0, 0)	0.003
Infrastructure	0 (0, 0)	0 (0, 0)	0 (0, 0)	0.6	0 (0, 0)	0 (0, 0)	0.003
**Radiology**							
Doctors	33 (14, 135)	49 (17, 132)	31 (14, 136)	0.2	106 (15, 289)	15 (14, 96)	<0.001
Nursing Staff	0 (0%)	0 (0%)	0 (0%)		0 (0%)	0 (0%)	
Other Staff	46 (19, 175)	64 (47, 187)	42 (19, 174)	0.037	120 (41, 560)	24 (19, 114)	<0.001

Medication	0 (0, 2)	0 (0, 0)	0 (0, 2)	0.2	1 (0, 15)	0 (0, 1)	<0.001
Medical Supplies	1 (0, 65)	11 (1, 48)	1 (0, 68)	0.10	23 (0, 387)	0 (0, 23)	<0.001
Infrastructure	42 (17, 213)	72 (38, 215)	42 (17, 211)	0.3	148 (33, 497)	23 (17, 138)	<0.001
**Surgery**							
Doctors	1,366 (902, 1,888)	1,125 (675, 1,344)	1,403 (928, 1,944)	<0.001	1,777 (1,294, 2,357)	1,248 (812, 1,670)	<0.001
Nursing Staff	0 (0%)	0 (0%)	0 (0%)		0 (0%)	0 (0%)	
Other Staff	1,088 (773, 1,521)	1,020 (642, 1,176)	1,102 (787, 1,535)	0.028	1,369 (1,064, 1,829)	1,008 (669, 1,334)	<0.001
Medication	38 (21, 56)	31 (20, 38)	40 (21, 59)	<0.001	46 (27, 66)	35 (19, 53)	<0.001
Medical Supplies	1,255 (742, 2,366)	971 (670, 1,851)	1,282 (767, 2,441)	0.015	767 (575, 1,055)	1,641 (969, 2,816)	<0.001
Infrastructure	1,437 (987, 1,878)	1,166 (876, 1,498)	1,467 (1,005, 1,893)	0.003	1,613 (1,209, 2,058)	1,378 (864, 1,832)	<0.001
**Anesthesiology**							
Doctors	874 (656, 1,087)	819 (579, 950)	878 (667, 1,110)	0.038	1,034 (849, 1,322)	808 (601, 988)	<0.001
Nursing Staff	0 (0%)	0 (0%)	0 (0%)		0 (0%)	0 (0%)	
Other Staff	582 (450, 741)	547 (410, 760)	583 (451, 739)	0.5	703 (561, 846)	543 (423, 671)	<0.001
Medication	67 (48, 83)	66 (49, 80)	67 (48, 83)	0.8	79 (63, 97)	62 (46, 76)	<0.001
Medical Supplies	176 (134, 223)	184 (141, 242)	175 (134, 222)	0.4	213 (172, 257)	165 (127, 203)	<0.001
Infrastructure	557 (429, 714)	470 (348, 562)	566 (436, 733)	<0.001	650 (528, 828)	513 (400, 654)	<0.001
**Laboratory**							
Doctors	123 (70, 178)	84 (52, 151)	125 (76, 181)	0.021	153 (97, 212)	105 (65, 162)	<0.001
Nursing Staff	0 (0%)	0 (0%)	0 (0%)		0 (0%)	0 (0%)	

Other Staff	212 (125, 310)	185 (99, 333)	213 (125, 306)	0.7	266 (172, 377)	181 (113, 286)	<0.001
Medication	0 (0, 130)	0 (0, 0)	0 (0, 146)	0.2	156 (0, 427)	0 (0, 0)	<0.001
Medical Supplies	421 (307, 589)	423 (268, 526)	421 (312, 596)	0.3	595 (416, 958)	371 (268, 499)	<0.001
Infrastructure	172 (104, 260)	119 (74, 214)	175 (108, 261)	0.013	218 (138, 317)	153 (92, 236)	<0.001
**Other**							
Doctors	20 (7, 35)	8 (8, 44)	20 (4, 31)	0.9	27 (10, 50)	18 (2, 27)	<0.001
Nursing Staff	4 (0, 7)	0 (0, 4)	4 (0, 7)	<0.001	5 (0, 8)	4 (0, 6)	0.003
Other Staff	109 (38, 195)	23 (4, 47)	119 (58, 206)	<0.001	145 (58, 259)	95 (27, 167)	<0.001
Medication	0.09 (0.00, 0.66)	0.09 (0.09, 0.19)	0.00 (0.00, 0.66)	>0.9	0.09 (0.00, 0.66)	0.09 (0.00, 0.66)	0.7
Medical Supplies	4 (0, 62)	91 (44, 125)	4 (0, 34)	<0.001	5 (2, 124)	4 (0, 35)	<0.001
Infrastructure	44 (22, 83)	20 (6, 41)	47 (25, 87)	<0.001	73 (37, 130)	40 (22, 68)	<0.001
